# Regulating Dielectric and Ferroelectric Properties of Poly(vinylidene fluoride-trifluoroethylene) with Inner CH=CH Bonds

**DOI:** 10.3390/polym10030339

**Published:** 2018-03-20

**Authors:** Yanan Zhang, Shaobo Tan, Jian Wang, Xiao Wang, Weiwei Zhu, Zhicheng Zhang

**Affiliations:** 1 Department of Applied Chemistry, MOE Key Laboratory for Nonequilibrium Synthesis and Modulation of Condensed Matter, School of Science, Xi’an Jiaotong University, Xi’an 710049, China; s1993125g@stu.xjtu.edu.cn (Y.Z.); shaobotan.1987@xjtu.edu.cn (S.T.); a1209113873@stu.xjtu.edu.cn (J.W.); yogurt_wx@stu.xjtu.edu.cn (X.W.); 2 Zhejiang Research Institute of Chemical Industry, No. 387 Tianmushan Road, Hangzhou 310000, China

**Keywords:** poly(vinylidene fluoride-trifluoroethylene), inner CH=CH bonds, regulating ferroelectric properties

## Abstract

Poly(vinylidene fluoride) (PVDF) based ferroelectric polymers have attracted considerable attention both academically and industrially due to their tunable ferroelectric properties. By pinning the conformation of the polymer chain and the ferroelectric phase physically or chemically, the ferroelectric behaviors of PVDF based polymers could be finely turned from normal ferroelectric into relaxor ferroelectric, anti-ferroelectric like, and even linear dielectric. Besides high energy electron irradiation and chemical copolymerization with the bulky monomers, in this work, an alternative strategy is presented to regulate the dielectric and ferroelectric performances of PVDF based ferroelectric polymer for the first time. CH=CH bonds with the desired content are inserted by a controlled dehydrofluorination reaction into a poly(vinylidene fluoride-trifluoroethylene) (P(VDF-TrFE)) copolymer (TrFE refers to trifluoroethylene) synthesized from the hydrogenation of P(VDF-CTFE) (CTFE refers to chlorothrifluoroethylene). The influence of the CH=CH bonds along with the fabrication conditions on the crystallization and ferroelectric relaxation of the resultant copolymers (referred to P(VDF-TrFE-DB)) was carefully characterized and discussed. The nonrotatable CH=CH bonds result in depressed dielectric and ferroelectric performances in the as-cast films by confining the orientation of ferroelectric grains in P(VDF-TrFE). The normal ferroelectric performance of P(VDF-TrFE) is turned into anti-ferroelectric like behavior in the resultant P(VDF-TrFE-DB). The cleavage of CH=CH bonds is responsible for the recovery of the ferroelectric behavior in the annealed samples. Uniaxial stretching favors the alignment of the polymer chain and ferroelectric domains, which may address the further regulated ferroelectric characters in the stretched samples.

## 1. Introduction

Poly(vinylidene fluoride) (PVDF) based fluoropolymers are typical semi-crystalline ferroelectric polymers exhibiting varied ferroelectric behaviors under electric fields depending on their chain conformation, ferroelectric, and crystalline phases [1,2,3,4]. Among them β-PVDF is the well-known normal ferroelectric polymer with excellent ferroelectric, piezoelectric, and pyroelectric performance, which has been well investigated over past decades in terms of sensors, ultrasonic devices, and wearable monitors [5,6,7,8]. Four crystal forms (α, β, δ, and γ) could be observed in PVDF, and β-PVDF has been fabricated under restricted conditions such as being stretched at low temperature with large extension ratios or by polarizing α-PVDF films under high electric fields [9,10]. β-PVDF ferroelectric material is also used in high precision measurements using new switching sensing devices, which compensate temperature drift [11,12]. By copolymerizing VDF with trifluoroethylene (TrFE) or tetrafluoethylene (TFE), the chain conformation of the resultant copolymers could be converted from the thermal dynamically stable TGTG form corresponding to α-phase of neat PVDF into the TTTG (an alternated *trans-gauche* conformation) and TTTT (a zigzag (all-*trans*) configuration) forms depending onto the molar content of TrFE and TFE in the resultant copolymers, which favors the formation of γ and β crystalline phases [13,14,15,16]. The copolymers with sufficient TrFE content (over 20 mol %) exhibit normal ferroelectric behavior and excellent piezoelectricity when polarized [17]. Besides, the crystalline transition, a *ferro*- to a *para*-electric (or Curie) transition would be detected in the copolymer depending on the TrFE content. The lowest *T*_C_ of P(VDF-TrFE) is about 60 °C, indicating that the materials are used in a ferroelectric phase and at an ambient temperature.

As for their various applications, PVDF based ferroelectric polymers with varied ferroelectric performances have gained continuous focus. On exposing the P(VDF-TrFE) films to a high energy electron beam, the large P(VDF-TrFE) ferroelectric grains are broken into small ones, and the same phenomenon occurs with crystalline domains [18]. The strong coupling force between the grains is eliminated owing to the reduced grain size and the improved distance between the grains. The normal ferroelectric behavior of P(VDF-TrFE) can be converted into relaxor ferroelectric characterized by the quickly reduced coercive field (*E*_c_) and remnant polarization (*P*_r_) at zero electric field. Alternatively, the normal ferroelectric behavior of P(VDF-TrFE) could be better pinned with third monomers embedded such as chlorotrifluoroethylene (CTFE), chlorofluoroethylene (CFE), and hexafluoropropylene (HFP). The addition of the bulky monomers can effectively tailor the sequential length of the all-trans P(VDF-TrFE) chain and reduce the size together with the overall content of crystalline phase [19,20]. *T*_C_ is decreased accordingly and 6–10 mol % CTFE or CFE is the optimum composition to achieve the best relaxor ferroelectric behavior at an ambient temperature in the resultant P(VDF-TrFE-C(T)FE) terpolymers [21].

Under increased external electric field, β-PVDF and P(VDF-TrFE) can exhibit anti-ferroelectric like behavior characterized with a double hysteresis on displacement-electric field loops (D–E) in a very narrow field range. This is associated with the alignment of isotropically dispersed dipole moments along the electric field. Recently, the anti-ferroelectric like behavior was realized in the polystyrene (PS) and poly(methyl methacrylate) (PMMA) grafted P(VDF-TrFE-CTFE) terpolymers over a wide electric field range [22]. In this case, the ferroelectric grains are pinned into a smaller size by bulky CTFE, which is well dispersed in the insulating PS or PMMA segment in the amorphous phase with high rigidity. These confined dipoles are gradually oriented by the electric field, and their disorientation is quickly completed as long as the electric field is removed thanks to the reduced coupling force among the ferroelectric grains [23]. As a result, the electric field required for polarization saturation is remarkably increased, and at zero electric field, a near zero Pr is observed. When a sufficient high content of PS or PMMA is grafted onto the terpolymer, the ferroelectric domains can no longer be oriented and only the linear D–E profiles are obtained [23,24].

Besides, electron beam irradiation and copolymerizing with third monomer strategies, we present an alternative way to tune the ferroelectric behaviors of the P(VDF-TrFE) copolymers. Into the P(VDF-TrFE) with a molar ratio of 80/20 mol %, the desired content of CH=CH bonds serving as defects is introduced by a controlled dehydrofluorination process catalyzed with Cu (0) and 2,2′-bipyridine (Bpy) as reported in the literature [25]. Different from the rotatable third monomer like CTFE and CFE, CH=CH bonds are nonrotatable and noncrystallizable in P(VDF-TrFE). They can not only tailor the sequential length of polar P(VDF-TrFE) chain but also confine their rotation along the electric field. That allows them to effectively tune the ferroelectric performances of P(VDF-TrFE) from normal ferroelectric to relaxor and anti-ferroelectric like behaviors in the as-cast P(VDF-TrFE-DB)s. More interestingly, CH=CH bonds show thermally unique cleavage properties, which cause the fine recovery of normal ferroelectric profiles in the annealed P(VDF-TrFE-DB)s for the eliminated confinement of CH=CH bonds on the ferroelectric polarization. The crosslinking induced by the breakage of CH=CH bonds allows the annealed samples to be uniaxially stretched in a large ratio. The stretching induced orientation of both polymer chain and ferroelectric grains favors the ferroelectric phase transition under external electric field

## 2. Materials and Methods

### 2.1. Materials

P(VDF-CTFE) 80/20 (with 20 mol % CTFE) was purchased from China Bluestar Chengrand Chemical Co. Ltd. (Beijing, China). Tetrahydrofuran (THF, Tianjin Reagents Co. Ltd., Tianjin, China, AR grade) was dried and distilled from sodium/benzophenone under nitrogen. 2,2′-Bipyridine (Bpy, Alfa Aesar, Shanghai, China, 99%), *N*-methylpyrrolidone (NMP, Tianjin Reagents Co. Ltd., Tianjin, China, AR grade), *N,N*-dimethylformamide (DMF, Tianjin Reagents Co. Ltd., Tianjin, China, AR grade) and acetone were purchased from Tianjin Reagents Co. Ltd. (Tianjin, China, AR grade) and used as received without further purification. Commercially available copper powder with a diameter of 75 μm was washed with dilute hydrochloric acid once and deionized water twice followed by drying in a N_2_ atmosphere before use and other chemicals were commercially available and used as received unless specified.

### 2.2. Synthesis of P(VDF-TrFE-DB) from P(VDF-TrFE) via SET-RE Process

The synthesis of P(VDF-TrFE-DB)s with the desired chemical composition was conducted following a single electron transfer radical elimination reaction (SET-RE) procedure of P(VDF-TrFE) [25], which was synthesized from full hydrogenation of P(VDF-CTFE) [21]. The typical procedure is as follows, 1.0 g P(VDF-TrFE) copolymer was dissolved in 100 mL DMSO completely in a N_2_ purged 250 mL two-necked flask before 0.19 g of copper powder (2.97 mmol) and 0.92 g of Bpy (5.89 mmol) were introduced. The reaction was conducted at 80 °C for 24 h in N_2_ atmosphere with vigorous stirring. The resultant mixture was precipitated in 3 wt % calcium chloride aqueous solution to remove the by-product HF. The raw product was dissolved in acetone followed by precipitating in methanol three times before it was dried at room temperature under reduced pressure. ^1^H and ^19^F NMR were applied to determine the exact composition of the objective copolymers. 

### 2.3. Film Fabrication under Varied Conditions, Instrumentation, and Characterization

All films were prepared via a solution casting process using the following procedure: 0.2 g of the resultant P(VDF-TrFE-DB) was dissolved in DMF (8 mL) under vigorous stirring overnight at room temperature. The films approximately 20 μm were obtained by casting the homogeneous solution onto clean glass slides, followed by heating at 50 °C until the solvent was evaporated thoroughly. Pristine P(VDF-TrFE) film was fabricated by the same process for comparison. The annealed films were prepared by heating the as-cast films at 150 °C for 4 h. The stretched films were obtained by uniaxially stretching the annealed films at room temperature to about 200% and 300% of their original length.

### 2.4. Instrumentation and Characterization

^1^H and ^19^F NMR spectra were recorded on a Bruker (Advance III) 400 MHz spectrometer in acetone-*d*_6_, using TMS as the internal standard. Fourier transform infrared (FTIR) spectroscopy of the films was obtained on a Tensor 27 (Bruker, Karlsruhe, Germany) with a resolution of 1–0.4 cm^−1^ in transmission mode. Differential scanning calorimetry (DSC) analysis was conducted on a NETZSCH DSC 200PC (NETZSCH Corporation, Bavaria, Germany). An amount of 8–12 mg of the sample was placed in an aluminum crucible and heated in a N_2_ atmosphere from 20 to 200 °C at a scanning rate of 10 °C·min^−1^. X-ray diffraction (XRD, RIGAKU D/MAX-2400, Rigaku Industrial Corporation, Osaka, Japan) was performed to characterize the crystal structures and crystallinity of the polymer films. The scanning range was from 10° to 60°, the step was 0.02 and the scanning rate was 10°·min^−1^. Stress-strain curves were obtained at a tensile speed of 10 mm·min^−1^ using a material testing machine CMT 6503 (Shenzhen Suns Technology Stock Co. Ltd., Shenzhen, China). Gold was coated onto both surfaces of the film with a thickness of 80 nm as electrodes using a JEOL JFC-1600 auto fine coater (JEOL Ltd., Tokyo, Japan). The dielectric properties were measured on an Agilent (4294A) precision impedance analyzer in a frequency range from 100 Hz to 10 MHz at 1 V. Broadband dielectric spectroscopy measurements were performed on a Novocontrol Concept 80 broadband dielectric spectrometer (Novocontrol Co. Ltd., Norwich, Germany) with temperature control. The applied voltage was 1 V with the frequency ranging from 0.01 Hz to 10 MHz and the temperature was varied from 20 °C, to 130 °C. D–E loops at room temperature were collected on a Premiere II ferroelectric tester from Radiant Technologies (Radiant Technologies Inc., Albuquerque, NM) at a frequency of 10 Hz.

## 3. Results and Discussion

### 3.1. Characterization and Chemical Composition of P(VDF-TrFE-DB)s

**Scheme 1:**
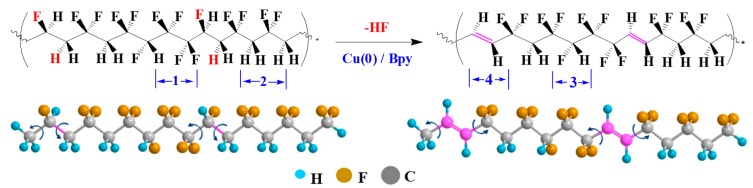
Chemical structure of P(VDF-TrFE-DB) synthesized from P(VDF-TrFE) by SET-RE mechanism.

**Figure 1:**
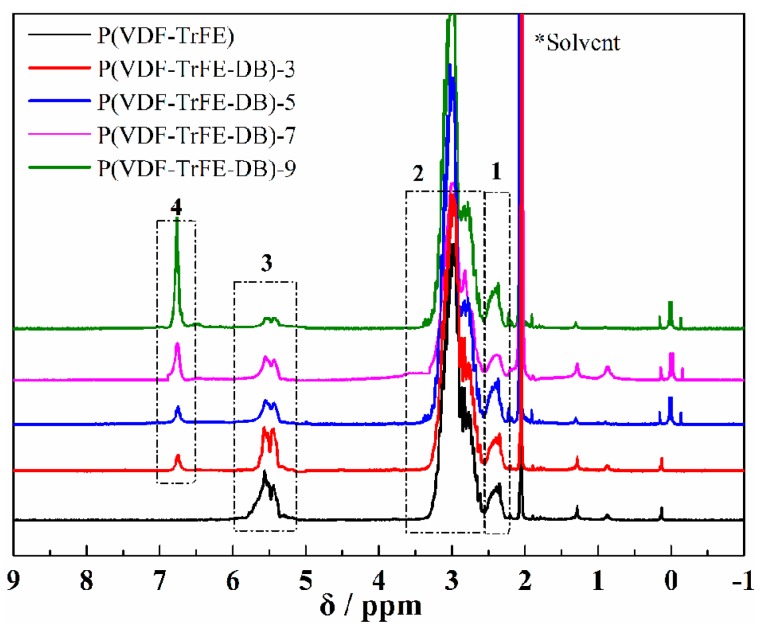
Typical ^1^H NMR spectra of P(VDF-TrFE) (80/20 mol %) and P(VDF-TrFE-DB)s with increased double bonds from 3 mol % to 9 mol %.

As illustrated in Scheme 1, P(VDF-TrFE-DB)s containing varied CH=CH double bonds were synthesized from P(VDF-TrFE). The chemical composition of the desired P(VDF-TrFE-DB)s was finely controlled and identified using ^1^H NMR as shown in Figure 1 and ^19^F NMR for comparison [26,27,28], which was previously well assigned. Comparing the ^1^H NMR spectra of P(VDF-TrFE), the new multiple signals (4) at 6.5–7.0 ppm of P(VDF-TrFE-DB)s from Figure 1 are identified as the protons on the double bonds (–CF_2_–CH=CH–CF_2_–) after the elimination of HF from (–CF_2_CH_2_–CFHCF_2_–). The integral of the signal increases with the content of double bonds, while the signal of TrFE decreases accordingly for the consumption of C–F bonds on TrFE. Since the dehydrofluorination involves one unit of VDF and one unit TrFE as suggested in Scheme 1, the resultant CF_2_–CH=CH–CF_2_ is treated as one CH=CH unit. Therefore, the chemical composition of P(VDF-TrFE-DB)s is calculated and listed in Table 1.

### 3.2. Crystalline Properties of P(VDF-TrFE-DB) Films Fabricated under Varied Conditions

**Figure 2:**
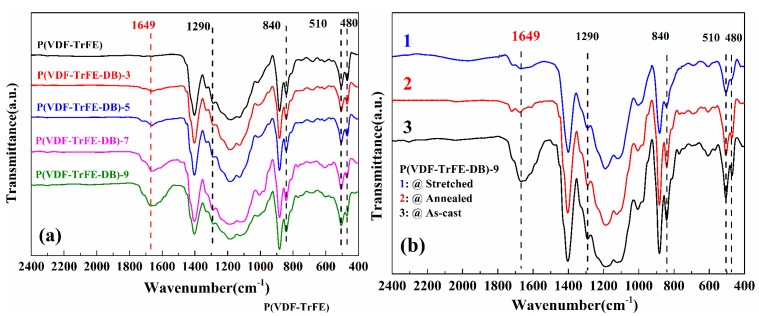
Fourier transform infrared (FTIR) spectra of the as-cast P(VDF-TrFE) (80/20 mol %) and P(VDF-TrFE-DB)s with different composition (**a**), and P(VDF-TrFE-DB)-9 with different treatment conditions (**b**).

The crystalline properties of P(VDF-TrFE-DB)s films fabricated under different conditions were characterized with XRD, FTIR, and DSC, respectively. As shown in Figure 2a, the new broad IR absorption appearing at 1649 cm^−1^ can be assigned to the CH=CH stretching of P(VDF-TrFE-DB), which is the major difference from the pristine P(VDF-TrFE). The FTIR absorbance at 1290 cm^−1^ corresponds to the *trans* isomer sequence with four or more than four units (characteristic of β crystal). The band at 840, 510 cm^−1^ is assigned to T_3_G conformation corresponding to β or γ phase. The absorption at 480 cm^−1^ is from the PVDF chain in amorphous phase. [16,29]. The slightly reduced intensity of these bands along with the rise of CH=CH content suggests that the β-phase crystalline contents have decreased [25]. Moreover, either annealing or stretching the as-cast films leads to significantly decreased intensity of these bands as shown in Figure 2b, where P(VDF-TrFE-DB)-9 films fabricated under different conditions are compared. Besides, the intensity of absorption corresponding to –CH=CH– at 1649 cm^−1^ of annealed and stretched samples is much lower than the as-cast film. That strongly shows that some of the CH=CH bonds have been involved in certain reactions during the annealing process at elevated temperature.

**Figure 3:**
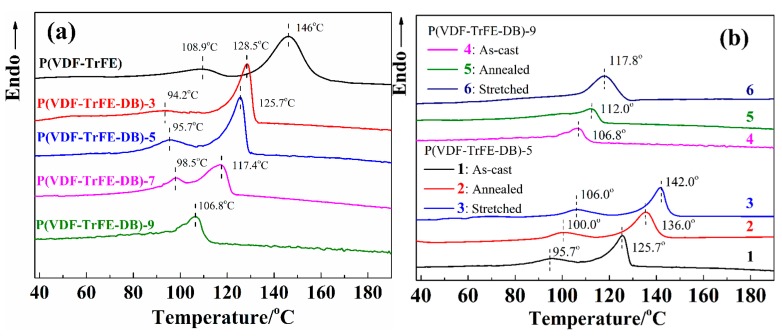
DSC curves of the as-cast P(VDF-TrFE) (80/20 mol %) and P(VDF-TrFE-DB)s with different composition (**a**), and P(VDF-TrFE-DB)-5 and P(VDF-TrFE-DB)-9 with different treatment conditions (**b**).

DSC was applied to indicate the *ferro*- to *para*-electric (F-P) phase transition together with the crystalline properties in P(VDF-TrFE) based ferroelectric polymers. As shown in Figure 3a, as the CH=CH content increases, the melting temperature (*T*_m_) of P(VDF-TrFE-DB)s is continuously decreased from 146 °C of pristine P(VDF-TrFE) to about 107 °C. The decreased *T*_m_ and the reduced melting enthalpy as listed in Table 2 strongly indicate that the content of the crystal phase is quickly reduced owing to the insertion of CH=CH bonds. The CH=CH bonds would interfere with the crystallization of the PVDF main chain instead of co-crystallizing with VDF units like TrFE, and the increasing CH=CH bonds may consume the TrFE units. Both effects lead to the decreased melting temperature and crystallinity. F-P transition (or Curie) temperature (*T*_C_) of all P(VDF-TrFE-DB)s is lower than P(VDF-TrFE) (ca. 108.9 °C), which could be attributed to the reduced rotation barrier of the P(VDF-TrFE) chain in the all-*trans* conformation after some TrFE units have been converted into CH=CHs serving as defects. As CH=CH content increases, *T*_C_ of P(VDF-TrFE-DB)s is gradually enhanced from 94.2 °C to about 98.5 °C at lower CH=CH content (less than 7 mol %), but the enthalpy is reduced. When CH=CH is 9 mol %, the *T*_C_ could hardly be detected because of combination with *T*_m_. The broad temperature range of *T*_C_ indicates that the insertion of CH=CH bonds results in a rather imperfect ferroelectric phase and the grains are dispersed in a large size range. It has been well discussed by Ohigashi and Hikosaka [30,31] using Gibbs free energy theory that the reduced crystallinity would result in enhanced Gibbs free energy of the paraelectric phase (Gp), thus increased *T*_C_ due to smaller crystal grains together with a more disordered arrangement of polymer chains. The interference of –CH=CH– bonds on the crystallization of the PVDF main chain and the consumption of the TrFE units may result in the overall content of β phase crystal. That may account for the lowered *T*_C_ of P(VDF-TrFE-DB)s compared to P(VDF-TrFE) and the gradually increased *T*_C_ as a function of CH=CH content. Based on the Gibbs free energy theory, annealing the P(VDF-TrFE) based ferroelectric polymers over their melting temperature would lead to increased *T*_m_ but reduced *T*_C_ for the elevated crystallinity, crystal grain size and the better ordered polymer chains. However, in the present P(VDF-TrFE-DB)s, annealing the as-cast films at 150 °C (ca. P(VDF-TrFE-DB)-5) results in both increased *T*_m_ and *T*_C_ as shown in Figure 3b and Table 2. As discussed above, the insertion of CH=CH bonds shows significant depression of the formation of ferroelectric and crystalline grains. Annealing at elevated temperature favors the crystallization of P(VDF-TrFE-DB). However, the F-P transition is shifted to higher temperature as well. Usually, stretching P(VDF-TrFE) based ferroelectric polymers would cause reduced *T*_C_ and improved *T*_m_ for the aligned ferroelectric domains and improved crystallinity induced by the polymer chain orientation. Differently, stretching the annealed P(VDF-TrFE-DB) samples at room temperature may further increase *T*_m_ and *T*_C_ as shown in Figure 3 and Table 2 synchronously. All these results suggest that more than simply physical change takes place during annealing the P(VDF-TrFE-DB)s. With respect to the intensity reduction of the CH=CH bonds in the FTIR results, we believe that some of the CH=CH bonds are broken at elevated temperature and cause the crosslinking in the annealed samples, which is discussed in detail in the Supplementary Materials.

**Figure 4:**
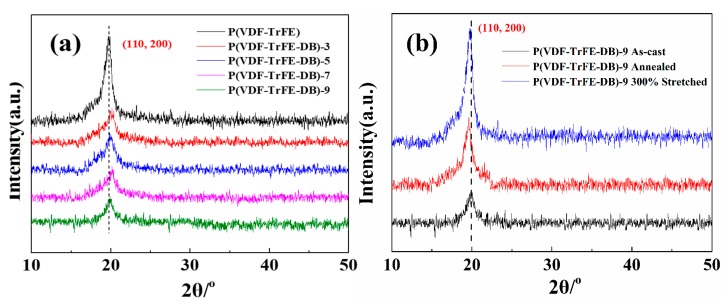
XRD spectra of the as-cast P(VDF-TrFE) (80/20 mol %) and P(VDF-TrFE-DB)s with different composition (**a**), and P(VDF-TrFE-DB)-9 with different treatment conditions (**b**).

XRD results were utilized to characterize the conformation from the crystallographic viewpoint as shown in Figure 4. The peak of initial P(VDF-TrFE) observed at 19.7° is associated with the all-trans conformation and represents the Bragg diffraction ((110)/(200)) of β phase [32,33]. As CH=CH content increases, the position of 2θ is slightly shifted to 20.3°, but the diffraction intensity is remarkably weakened. As suggested by Bragg equation 2d sin θ = nλ (n = 1, 2, …), CH=CH may decrease the distance (d) between P(VDF-TrFE) chains for the reduced steric bulk, which is rather different from the bulky monomers, such as CTFE, CFE, and HFP [34,35,36]. Meanwhile, the insertion of CH=CH bonds may damage the crystallization of P(VDF-TrFE) chains due to the increased rotation capability of CH–CF_2_ σ-bonds adjacent to CH=CH bonds. 

After annealing at 150 °C and followed by stretching the annealed film at room temperature, the intensity of the diffraction peak in P(VDF-TrFE-DB)-9 films is improved, while the angle is finely maintained as suggested in Figure 4b. This means that annealing and stretching lead to the increased crystallinity due to the cleavage of CH=CH bonds as discussed above. However, the distance between P(VDF-TrFE) chains is invisibly changed. Correspondingly, the confinement of CH=CH bonds on the crystallization is eliminated, which is responsible for the elevated crystallinity and *T*_m_. However, the formation of crosslinking among polymer chains increases the transition energy barrier of the ferroelectric domains as well, which may address the elevated *T*_C_ as discussed above.

### 3.3. Dielectric Properties under Low Electric Field

**Figure 5:**
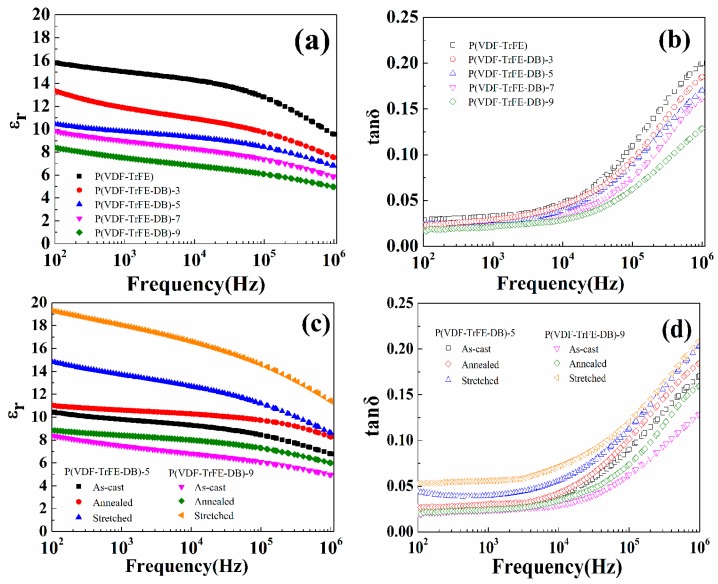
ε_r_ and tan δ of the as-cast P(VDF-TrFE) (80/20 mol %) and P(VDF-TrFE-DB)s (**a**,**b**), and comparison for P(VDF-TrFE-DB)-5 and P(VDF-TrFE-DB)-9 with different treatment conditions (**c**,**d**).

The dielectric properties of P(VDF-TrFE-DB)s under low electric field (bias voltage = 1 V) was determined by measuring the parallel capacitor and dissipation factor (Cp–D) as a function of frequency. The calculated dielectric permittivity (ε_r_) and loss (tan δ) are presented in Figure 5. As the double bonds increase, ε_r_ of P(VDF-TrFE-DB)s is continuously depressed. Under low electric field, the dielectric response of PVDF based ferroelectric polymers is mostly attributed to both the dipole alignment in the amorphous phase in the rubber state, and the weak swing of the dipoles in the micro-crystalline grains [37,38]. It is well known that the dielectric response of semi-crystalline PVDF-based fluoropolymers at frequencies below 100 kHz is attributed to both the dipole alignment in the amorphous phase in the rubber state and the weak swing of the dipoles in the microcrystal grains. As frequency increases from 100 kHz to 1 MHz, the contribution of crystal phase weakens gradually because of its slower relaxation characterized by a sharp increase of dielectric loss at this frequency range. When the frequency is over 1 MHz, the dielectric response only comes from molecular motions including the micro-Brownian motion of non-crystalline chain segments (β relaxation) and the molecular motion on the amorphous-crystalline interfaces. As frequency increases, the dielectric response of the dipoles continuously decreases, which leads to the reduced dielectric constant. The introduction of CH=CH bonds could effectively pin the length of the polymer chain in all-*trans* conformation, thus the size of the ferroelectric domains is reduced, which favors the dielectric response of the ferroelectric phase. However, the CH=CH bonds could not rotate like C–C single bonds as suggested by Scheme 1, which may confine the orientation of the polymer chain in TTT conformation between two CH=CH bonds along the electric field and depress the permittivity. This is rather different from the insertion of rotatable third monomer with large steric bulk such as CTFE, HFP, and CFE. Besides, the overall content of the ferroelectric phase is reduced as more CH=CH bonds are inserted, which is responsible for the reduced permittivity as well. Accordingly, the reduced ferroelectric phase both in content and size and the depressed rotation capability of ferroelectric grains are responsible for the depressed dielectric loss as well, as presented in Figure 5b. Especially, the ferroelectric relaxation inducing high loss at frequency ranging from 10 kHz to 1 MHz is dramatically reduced with the content of CH=CH bonds.

Annealing and stretching uniaxially have been widely applied to improve the dielectric performances of PVDF based ferroelectric polymers. After annealing, the permittivity of P(VDF-TrFE-DB) films is slightly improved as shown in Figure 5c,d. Taking P(VDF-TrFE-DB)-5 and P(VDF-TrFE-DB)-9 as instances, at low frequency, the improvement is rather small. As the frequency increases, the permittivity increment of the annealed samples is enlarged. This suggests the mobility of the ferroelectric grains is increased, which might be ascribed to the breakage of CH=CH bonds as discussed above. As a result, the dielectric loss at high frequency corresponding to the ferroelectric relaxation is increased as well. Stretching the annealed films uniaxially leads to significantly increased dielectric performances as indicated in Figure 5c,d. For example, at 1 kHz, ε_r_ of the stretched P(VDF-TrFE-DB)-5 and P(VDF-TrFE-DB)-9 films is improved to 14.8 and 19.0 from 11.3 and 8.8 of the annealed samples, respectively. This could be mainly attributed to the orientation of ferroelectric grains along the orientation direction, which could improve the dielectric response of the ferroelectric grains to the external field as reported previously [39,40]. The increased tan δ at low frequency and well maintained tan δ at high frequency could finely confirm the conclusion that the improved permittivity is mostly from the mobility improvement instead of the content increment of the ferroelectric phase. Meanwhile, when the crystallinity of the annealed sample is lower (ca. P(VDF-TrFE-DB)-9), stretching may increase the crystallization as discussed above and favor the formation of the ferroelectric domain as well. This may address the more significantly improved dielectric properties in the stretched P(VDF-TrFE-DB)-9 film compared to that of P(VDF-TrFE-DB)-5. In general, the slightly improved dielectric constant and loss induced by annealing or stretching could be ascribed to the increased crystallinity of P(VDF-TrFE-DB)s. Annealing induced breakage of CH=CH bonds and stretching resulting in orientation of the ferroelectric grains could weaken the confinement of the double bonds on the polymer segments in the all-trans conformation. This may account for the dramatically improved dielectric constant in the uniaxially orientated P(VDF-TrFE-DB) films.

### 3.4. F-P Transition Behaviors in P(VDF-TrFE-DB)s Copolymers

**Figure 6:**
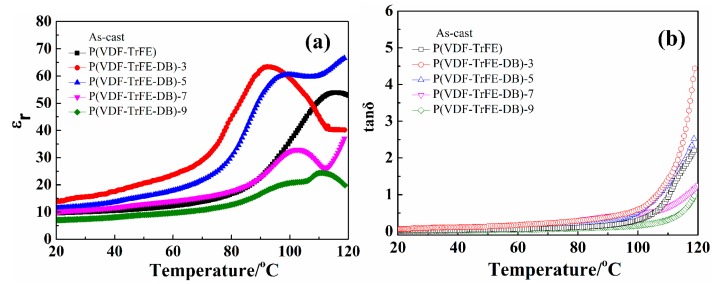
Dielectric permittivity (**a**) and dielectric loss (**b**) as a function of temperature at 1 kHz for the as-cast P(VDF-TrFE) and P(VDF-TrFE-DB)s.

In order to explore the F-P transition behavior of P(VDF-TrFE-DB)s, the temperature dependence of the dielectric constant and loss as a function of frequency for P(VDF-TrFE-DB)s with different chemical composition and pristine P(VDF-TrFE) were investigated. As shown in Figure 6a,b, the permittivity and tan δ of different samples measured at 1 kHz are presented as a function of temperature. For the pristine P(VDF-TrFE), a mono permittivity peak is obtained at about 115 °C corresponding to the F-P transition, which agrees well with the previously reported results [41]. The insertion of 3 mol % CH=CH causes a reduced F-P temperature to about 94 °C. Further increasing the CH=CH content leads to gradually increased F-P temperature to about 100 °C, and two peaks at 90 °C and 110 °C are observed in the sample P(VDF-TrFE-DB)-9. At *T*_C_, the maximum ε_r_ observed in P(VDF-TrFE-DB)-3 is 65, which is larger than P(VDF-TrFE) (ca. 55). As the CH=CH content increases to 9 mol %, the maximum ε_r_ is gradually reduced to about 20. The insertion of low content of CH=CH bonds may reduce the size of ferroelectric grains, and then improve the dielectric response at *T*_C_. However, too many CH=CH bonds inserted into the P(VDF-TrFE) chain would significantly depress the content of the ferroelectric phase due to the impediment of rigid CH=CH bonds on the crystallization of the P(VDF-TrFE) main chain. The separated *T*_C_ peaks in P(VDF-TrFE-DB)-9 suggest that the varied F-P transition barrier results from the ferroelectric grains of different sizes.

**Figure 7:**
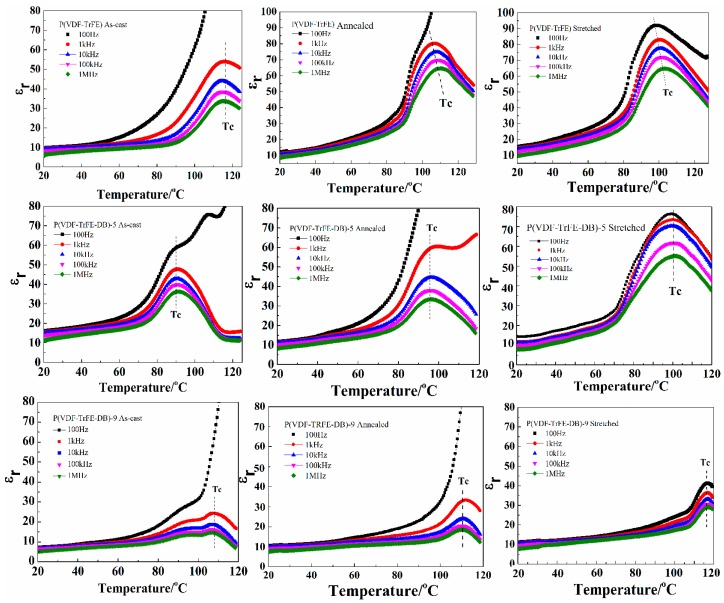
Influence of annealed and stretched treatment on the permittivity of P(VDF-TrFE), P(VDF-TrFE-DB)-5, and P(VDF-TrFE-DB)-9.

Figure 7 presents the influence of annealing and stretching on the dielectric spectrum of P(VDF-TrFE-DB)s as a function of temperature. For the neat P(VDF-TrFE), annealing at 150 °C leads to significantly increased permittivity at *T*_C_ from 55 to 80 at 1 kHz. Interestingly, the normal ferroelectric behavior has been turned into relaxor ferroelectric since the maximum permittivity shows frequency dependence in the annealed samples, which is rather different from the as-cast samples [38]. Stretching the P(VDF-TrFE) films uniaxially results in a slightly increased dielectric constant and further reduced *T*_C_, where the maximum permittivity is observed. This means the orientation of ferroelectric grains favors the dielectric response under low electric field, which has been well reported in PVDF based ferroelectric polymers [42]. For P(VDF-TrFE-DB)s, annealing at elevated temperature may dramatically increase the permittivity at *T*_C_ as shown in Figure 7. Stretching the annealed films could further enhance ε_r_. As discussed above, annealing the sample at elevated temperature may cause the breakage of partial CH=CH bonds, which would reduce the impediment effect on the crystallization of the P(VDF-TrFE) main chain and favor the improvement of ε_r_. Besides, for P(VDF-TrFE) ferroelectric polymers, annealing at elevated temperature is favorable for the formation of ferroelectric domains thus the reduced *T*_C_. Abnormally, annealing and stretching lead to increase *T*_C_ in the P(VDF-TrFE-DB)s, such as P(VDF-TrFE-DB)-5, whose *T*_C_ is increased from 85 °C to 95 °C for the annealed sample and 100 °C for the stretched sample, which is consistent with the DSC results. This is most likely associated with the breakage of partial CH=CH bonds and the formation of more badly ordered ferroelectric grains. Meanwhile, the crosslinking constructed between polymer chains would help to shift the *T*_C_ of P(VDF-TrFE-DB) to even higher temperature. Most notably, annealing and stretching do not change the normal ferroelectric nature of P(VDF-TrFE-DB), which is rather different from the pristine P(VDF-TrFE). This means the pristine P(VDF-TrFE) in the absence of CH=CH bonds is more like a high temperature relaxor ferroelectric performance. However, the insertion of rigid CH=CH bonds may stabilize the normal ferroelectric performance. The crosslinking induced by annealing at elevated temperature favors the normal ferroelectric behavior as well, and even uniaxial stretching is not able to change the normal terpolymers such as P(VDF-TrFE-C(T)FE) with a bulky third monomer as kink.

### 3.5. Ferroelectric Properties under High Electric Fields

**Figure 8:**
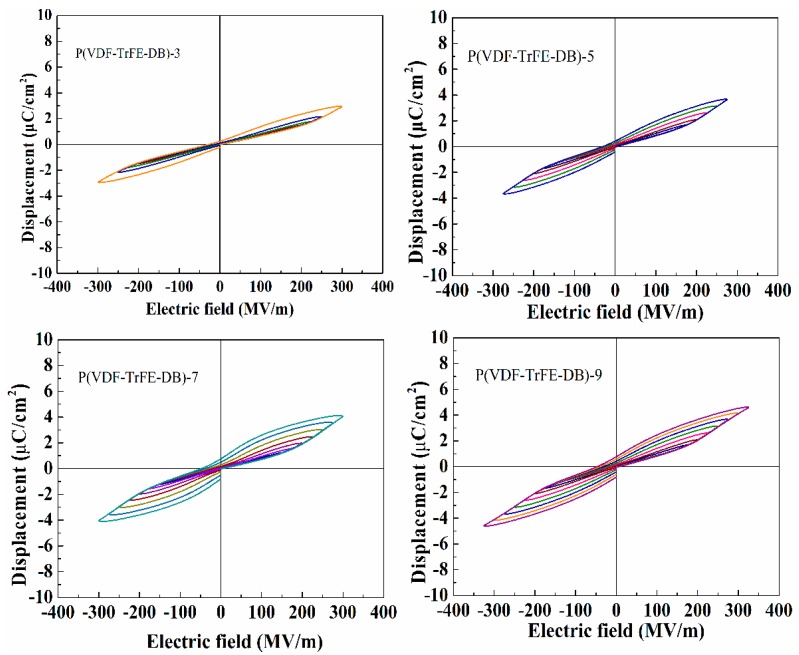
D–E loops of P(VDF-TrFE-DB)s films from solution cast at 50 °C.

**Figure 9:**
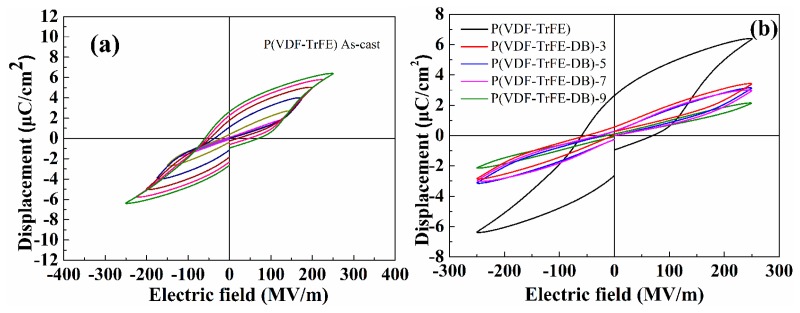
Bipolar D–E loops of P(VDF-TrFE) (20 mol % TrFE) film from solution cast at 50 °C (**a**) and comparison of D–E loops for P(VDF-TrFE-DB)s at 10 Hz and 250 MV·m^−1^ (**b**).

The effect of the CH=CH structure on the ferroelectric performance of P(VDF-TrFE) was investigated by the D–E hysteresis loops, measuring under standard bipolar and unipolar electric fields at a frequency of 10 Hz. The electric field is stepwise enhanced from zero at an increment of 25 MV·m^−1^ until the films are electrically broken down. As shown in Figure 8, the insertion of CH=CH as a rigid kink in the P(VDF-TrFE) chain could gradually confine the orientation of the ferroelectric grains. As a result, the normal ferroelectric behavior of pristine P(VDF-TrFE) is turned into anti-ferroelectric like behavior characterized with the propeller-shaped D–E loops in a broad electric field range [43,44]. As compared in Figure 9, the hysteresis loops of P(VDF-TrFE-DB)s with CH=CH content from 3 to 9 mol % show narrowed D–E loops under a consistent electric field, including the depressed *P*_m_, *P*_r_, *E*_c_ and hysteresis. Interestingly, the anti-ferroelectric like D–E loops are rather similar to that reported in polystyrene(PS) and Polymethyl methacrylate(PMMA) grafted P(VDF-TrFE-CTFE) terpolymers [22]. Apparently, that should be attributed to not only the pinning effect of CH=CH on the size of the ferroelectric domain but also the nonrotatable nature of the CH=CH bonds.

**Figure 10:**
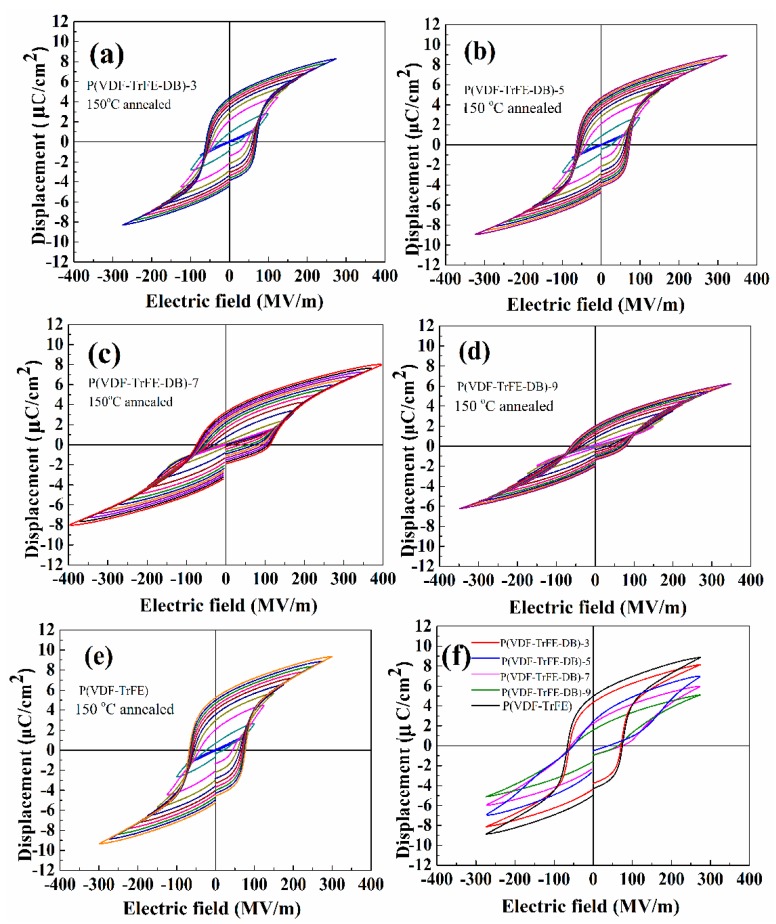
Bipolar D–E loops of P(VDF-TrFE-DB)s with different composition and P(VDF-TrFE) annealed at 150°C (**a**–**e**), and comparison of D–E loops at 275 MV·m^−1^ (**f**).

Annealing the P(VDF-TrFE-DB)s at elevated temperature (150 °C) leads to significant change in the D–E loops as shown in Figure 10, where the D–E loops of annealed P(VDF-TrFE) and P(VDF-TrFE-DB)s under the same electric filed are presented. Rather different from the anti-ferroelectric like behavior in the as-cast samples, the annealed samples exhibit normal ferroelectric behavior with significantly increased *P*_m_ and *P*_r_. The narrow D–E profiles could only be detected under an electric field below 50 MV·m^−1^ As discussed above, the anti-ferroelectric like characteristics in as-cast films could be ascribed to the tailoring and confining effects of nonrotatable CH=CH bonds on the ferroelectric phase of P(VDF-TrFE) in TTT conformation. However, annealing the P(VDF-TrFE-DB)s at elevated temperature would lead to the cleavage of CH=CH bonds thus the crosslinking of the films. The conversion of nonrotatable CH=CH bonds into rotatable CH-CH bonds would quickly release the confined ferroelectric response of P(VDF-TrFE) grains. As a consequence, the normal ferroelectric behavior is recovered with the breakage of CH=CH bonds. However, as CH=CH content increases, both Pr and Pm values under the same electric field are continuously reduced while *E*_c_ is improved. This could be attributed to the lowered TrFE content and the reduced impedance of the remained CH=CH bonds and the crosslinking points among the polymer chains. Both effects would certainly reduce the ferroelectric grain in size and the overall content. Therefore, anti-ferroelectric like behavior could only survive in the samples with higher CH=CH content. The cleavage of CH=CH bonds is responsible for the recovery of the normal ferroelectric profiles in P(VDF-TrFE-DB)s.

**Figure 11:**
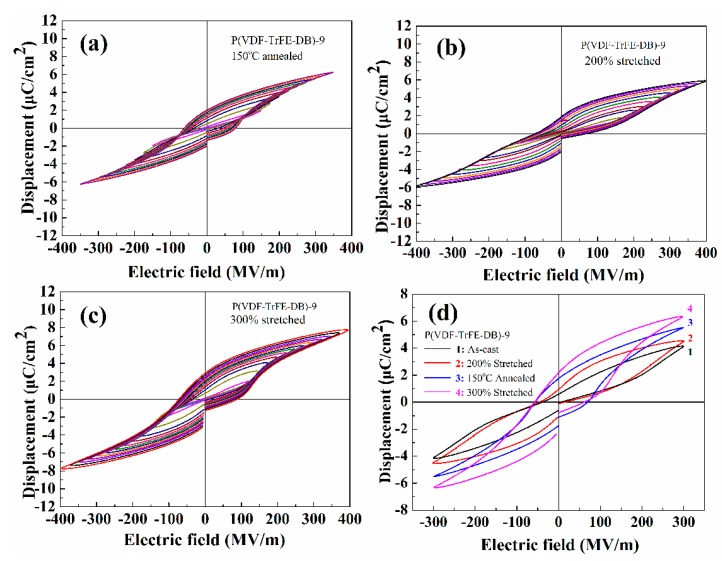
The bipolar D–E loops of P(VDF-TrFE-DB)-9 annealed at 150°C (**a**), P(VDF-TrFE-DB)-9 at room temperature stretched 200% (**b**), stretched 300% (**c**), and comparison of D–E loops for P(VDF-TrFE-DB)-9 treated under different conditions at 10 Hz and 300 MV·m^−1^ (**d**).

To show how the uniaxial stretching affects the ferroelectric performances of the P(VDF-TrFE-DB)s, the annealed P(VDF-TrF-DB)-9 films are taken as instances, which are stretched with different ratios (200% and 300%). As shown in Figure 11, as the stretching ratio increases, propeller-shaped double hysteresis loops appear gradually. This means the ferroelectric behavior of annealed P(VDF-TrFE-DB)-9 is gradually converted into anti-ferroelectric like profiles under an electric field ranging from 150 to 350 MV·m^−1^. Under a sufficiently high electric field, the double hysteresis loops are gradually turned into normal ferroelectric profile in all the samples. It has been well discussed that stretching uniaxially would cause the orientation of the polymer chains and ferroelectric domains along the orientation direction, which favors the orientation and disorientation of dipoles along the electric field. This would address the improved polarization under the same electric field, the reduced *E*_c_ and *P*_r_ [22,41].

**Figure 12:**
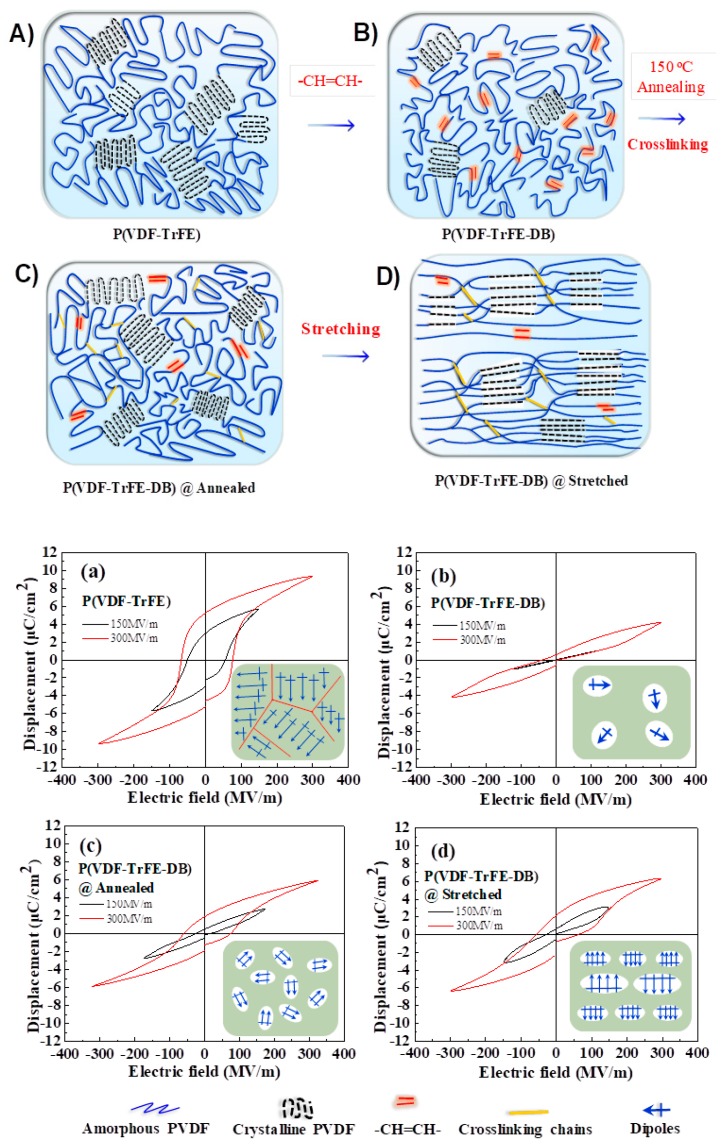
Evolution of ferroelectric phase in P(VDF-TrFE) and P(VDF-TrFE-DB)s under different processing conditions.

In general, the evolution of the ferroelectric phase transition undergoes the following process with the insertion of CH=CH bonds into P(VDF-TrFE) as suggested in Figure 12. First of all, the incorporation of nonrotatable CH=CH bonds would chop the sequence length of the P(VDF-TrFE) chain and depress the ferroelectric response under electric field. The normal ferroelectric P(VDF-TrFE) is gradually converted into anti-ferroelectric like profiles in P(VDF-TrFE-DB)s. However, CH=CH bonds would be broken at the elevated temperature, which would release the depressed ferroelectric grains which is responsible for the recovered normal ferroelectric behavior in annealed P(VDF-TrFE-DB)s. Stretching the annealed films would cause the alignment of the ferroelectric grains and polymer chains. This may account for the anti-ferroelectric behavior under low electric field (below 150 MV·m^−1^) and normal ferroelectric profiles under high electric field (over 300 MV·m^−1^) observed in the stretched P(VDF-TrFE-DB) films.

## 4. Conclusions

In summary, CH=CH bonds were successfully introduced into P(VDF-TrFE) (80/20) main chain via SET-RE. The nonrotatable and non-crystallizable natures of CH=CH bonds are responsible for the depressed dielectric and ferroelectric response in the as-cast films. The thermal cleavage of CH=CH bonds and the releasing of a confinement effect on the ferroelectric P(VDF-TrFE) may account for the recovery of high dielectric and ferroelectric behaviors in annealed P(VDF-TrFE-DB) samples. Uniaxial stretching would cause the orientation of both polymer chains and ferroelectric grains along the stretching direction, which favors the ferroelectric response under the same electric field. By regulating the CH=CH bonds in the ferroelectric P(VDF-TrFE) main chain, the dielectric and ferroelectric behavior of P(VDF-TrFE) could be facilely tuned. Especially, the thermal self-cleavage character of CH=CH bonds in P(VDF-TrFE-DB)s offers a robust strategy to tune the ferroelectric behavior of PVDF based ferroelectrics.

## Figures and Tables

**Table 1:** Abbreviations for the materials and chemical components.

Abbreviations	Chemical Components (VDF/TrFE/CH=CH in mol %)
P(VDF-TrFE-DB)-3	77/17/3
P(VDF-TrFE-DB)-5	75/15/5
P(VDF-TrFE-DB)-7	73/13/7
P(VDF-TrFE-DB)-9	71/11/9

**Table 2:** The crystallization characterization data of the copolymer films.

Sample	*T*_C_ (°C)	△*H*_c_ (J/g)	*T*_m_ (°C)	△*H*_m_ (J/g)
P(VDF-TrFE-DB)-5 as-cast	97.5	5.82	125.7	7.31
P(VDF-TrFE-DB)-5 annealed	100.0	5.86	136.0	7.49
P(VDF-TrFE-DB)-5 stretched	106.0	6.04	142.0	8.03
P(VDF-TrFE-DB)-9 as-cast	106.8	N.A	106.8	4.08
P(VDF-TrFE-DB)-9 annealed	112.0	N.A	112.0	7.26
P(VDF-TrFE-DB)-9 stretched	117.8	N.A	117.8	10.65

## Supplementary Materials

The following are available online at http://www.mdpi.com/2073-4360/10/3/339/s1, Scheme S1: Possible thermal crosslinking pathways of P(VDF-TrFE-DB)s at elevated; Figure S1: Comparison of the solubility of P(VDF-TrFE-DB)-9 and P(VDF-TrFE)(80/20) processed at different temperatures (**a**), time dependence of the gel fraction of P(VDF-TrFE-DB)-9 stirred at 110 °C, 150 °C about 6 h (**b**); Figure S2: Stress-strain curves of P(VDF-TrFE-DB)-9 loadings under room temperature.
