# Supplementary material for: Regulating Dielectric and Ferroelectric Properties of Poly(vinylidene fluoride-trifluoroethylene) with Inner CH=CH Bonds
Source: Polymers (Basel). 2018 Mar 20;10(3):339. doi: 10.3390/polym10030339 (PMC6415107; doi:10.3390/polym10030339)
Supplement: Supplementary file 1 [file polymers-10-00339-s001.pdf]

Supplementary Materials: Regulating Dielectric and Ferroelectric Properties of Poly(vinylidene fluoride-trifluoroethylene) with Inner CH=CH Bonds

Yanan Zhang, Shaobo Tan, Jian Wang, Xiao Wang, Weiwei Zhu and Zhicheng Zhang

1. Possible thermal crosslinking mechanism of P(VDF-TrFE-DB) copolymers

Based on the discussion in main text, the cleavage of CH=CH bonds and crosslinking in the P(VDF-TrFE-DB) films could be certainly determined during the thermal treatment. Since no radical initiator is added, the thermal crosslinking of P(VDF-TrFE-DB)s might be in two possible ways. One is the direct cleavage of CH=CH bonds to form *CH-CH* radicals followed by the addition toward another CH=CH bond or coupling reactions between two *CH-CH* radicals, which is presented in Scheme S1(a). The other one is associated with the O_2 in the atmosphere. As described in Scheme S1(b), the insertion of O_2 into CH=CH bonds to form peroxide species and the breakage of -O-O- bonds in C-O-O-C are most likely to take place for the generation of C-O* radicals. Subsequently, C-O* would attack the other adjacent CH=CH bonds. The newly created C-O-C or C-C covalent bonds among different polymer chains are responsible for the gelation of the P(VDF-TrFE-DB)s, thus the improved mechanical performances and depressed solubility in good solvents. Unfortunately, C-O-C covalent bonds could not be detected by FTIR in the annealed films, which could not exclude the possibility of the O_2 mediated crosslinking for the low concentration of C-O-C bonds.

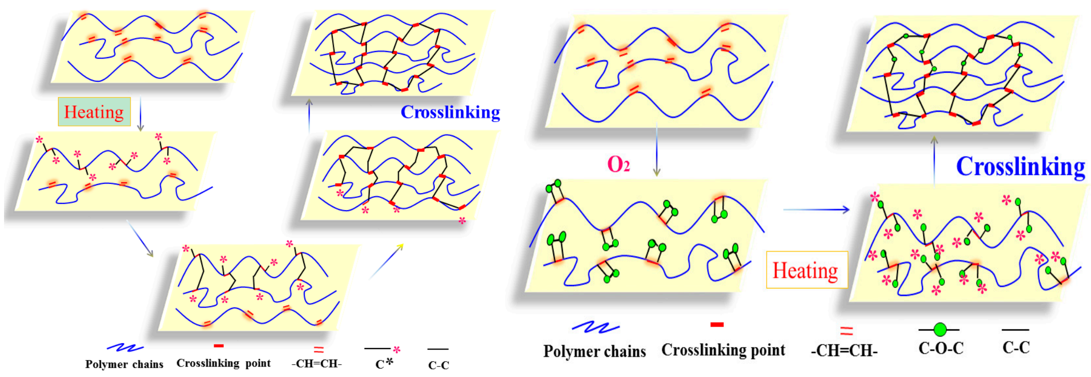

(a) (b)

Scheme S1. Possible thermal crosslinking pathways of P(VDF-TrFE-DB)s at elevated temperature.

2. Solubility and the crosslinking speed of the annealed films in acetone

To confirm the annealed P(VDF-TrFE-DB) films at elevated temperature might result into the breakage of CH=CH bonds as discussed above, P(VDF-TrFE-DB) films with a dimension of 70mm×20mm×0.02mm are fabricated for the thermally initiated gelation investigation. After heated at increased temperature from 70 to 150 ^{o}C with an increment step of 20 ^{o}C for 24 h, the as-cast films are put in acetone for 24 h with vigorous stirring. The sample annealed at

70 °C could be dissolved in acetone, but the solution loses the transparency. Annealed at temperature over 90 °C, the films are totally insoluble in acetone as shown in Figure S1(a). For comparison, even annealing at 150 °C causes invisible gelation in the pristine P(VDF-TrFE), indicating P(VDF-TrFE) is thermally stable. To quantitatively evaluate the gelation process of the films, as a function of annealing time, the mass ratio of insoluble part (m_1/m_0) of the P(VDF-TrFE-DB)-9 sample annealed at 110 and 150 °C is presented in Figure S1 (b), where m_1 and m_0 are referring to the absolute mass of the dried films after and before extracted with acetone. At elevated temperature, the as-cast films are gelled quickly without any extra free radical initiator input. 30 wt% at 110 °C and over 40 wt% at 150 °C of the films are insoluble in

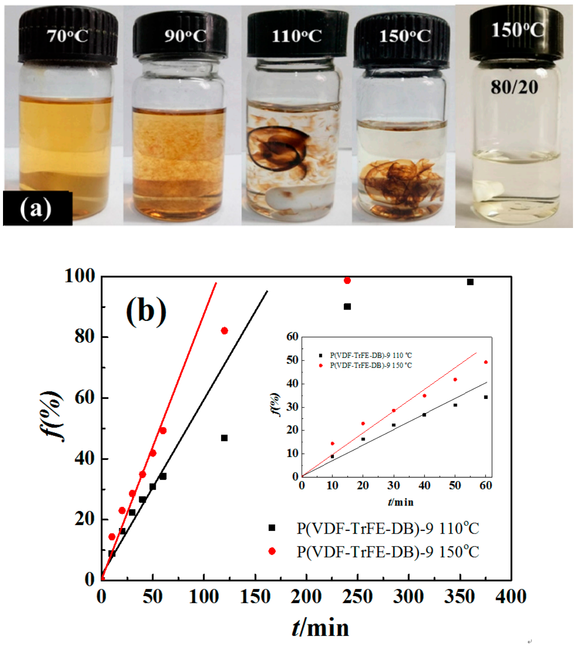

Figure S1. Comparison of the solubility of P(VDF-TrFE-DB)-9 and P(VDF-TrFE) (80/20) processed at different temperatures (a), time dependence of the gel fraction of P(VDF-TrFE-DB)-9 stirred at 110 °C, 150 °C about 6 h (b).

1 hour. At 150 °C, the insoluble fraction is increased linearly in the initial 2 h, while at 110 °C, the linear increasing tendency could be well maintained in the initial 4 h. That suggests the breakage of the CH=CH bonds is very likely following the first order reaction kinetics. After 4 h at 150 °C or 6 h at 110 °C, the samples are completely gelled suggesting the CH=CH bonds are rather sensitive to the elevated temperature.

3. Stress-strain profiles of as-cast and annealed P(VDF-TrFE-DB) films

The thermal crosslinking of P(VDF-TrFE-DB) films leads to not only the solubility reduction but also the mechanical performance improvement. As shown in Figure S2, the stress-strain curves of the P(VDF-TrFE-DB)-9 films before and after annealed at 150 °C for 4 h are presented. The as-cast P(VDF-TrFE-DB)-9 film with low crystallinity is showing a typical plastic stretching profile with a low yield strength (about 5 MPa) and high elongation ratio (over 100%). The reduced stress during the elongation is ascribed to the quickly decreased specimen dimensions during stretching. After annealed at 150 °C, the yield strength is observed as 9 MPa, which is almost doubled the value of the as-cast film. Meanwhile, the elongation at breakage is reduced to about 50%, which means the covalent crosslinking

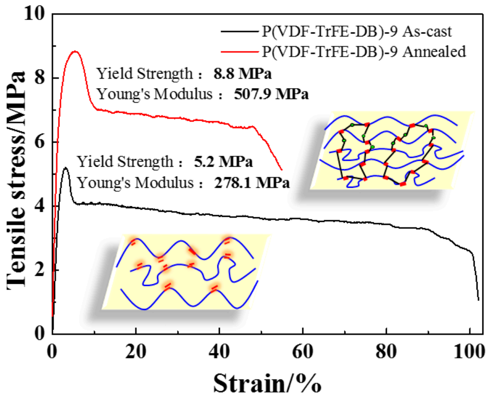

Figure S2. Stress-strain curves of P(VDF-TrFE-DB)-9 loadings under room temperature.

Causes significant restriction on the elongation scale of the film. The annealed P(VDF-TrFE-DB)-9 film possesses nearly doubled Young's modulus (508 MPa) of the as-cast film (278 MPa), which could confirm the thermal induced gelation as well.

In general, during annealing the P(VDF-TrFE-DB) films at elevated temperature, chemical crosslinking does take place in certain way, which is definitely related to the cleavage of CH=CH bonds. That may address the change taking place in the films after annealing, including the reduced solubility in good solvents, the improved modulus and reduced elongation, and the dielectric properties as discussed in the main text.
